# Tigecycline use in serious nosocomial infections: a drug use evaluation

**DOI:** 10.1186/1471-2334-10-287

**Published:** 2010-09-29

**Authors:** Matteo Bassetti, Laura Nicolini, Ernestina Repetto, Elda Righi, Valerio Del Bono, Claudio Viscoli

**Affiliations:** 1 Infectious Diseases Division, San Martino Hospital and University of Genoa School of Medicine, Genoa, Italy

## Abstract

**Background:**

Tigecycline is a novel antibiotic with activity against multidrug resistant bacteria. The aim of this study was to assess the efficacy of tigecycline use in serious hospital-acquired infections (HAI)

**Case presentation:**

Prospective observational study of tigecycline use was conducted in a 1500 beds university hospital. From January 1, 2007 and January 31, 2010, 207 pts were treated with tigecycline for the following indications: intra-abdominal, pneumonia, bloodstream and complicated skin and soft tissue infections and febrile neutropenia. The therapy was targeted in 130/207 (63%) and empirical in 77/207 (37%) patients. All bacteria treated were susceptible to tigecycline. Median duration of tigecycline therapy was 13 days (range, 6-28). Clinical success was obtained in 151/207 (73%) cases, with the highest success rate recorded in intra-abdominal infections [81/99 (82%)]. Microbiological success was achieved in 100/129 (78%) treated patients. Adverse clinical events were seen in 16/207 patients (7.7%):

**Conclusions:**

Considering the lack of data on tigecycline for critically ill patients, we think that the reported data of our clinical experience despite some limitations can be useful for clinicians.

## Background

The management of hospital-acquired bacterial infections is becoming a significant challenge for health care providers because of the increased prevalence of multidrug-resistant (MDR) bacteria like methicillin-resistant *Staphylococcus aureus *(MRSA), vancomycin-resistant *Enterococcus spp*. (VRE), *Acinetobacter baumannii*, *Klebsiella pneumoniae*, carbapenemase -producing Enterobacteriaceae, and extended-spectrum beta-lactamase (ESBL)-producing Enterobacteriaceae. Increased morbidity and mortality, duration of hospitalization, and medical care costs are all associated with MDR organisms [1]. Delay of appropriate empiric antimicrobial therapy is known to increase morbidity and mortality among affected patients and inadequate therapy has been found to be associated with excess mortality and increased duration of hospitalization [2]. There is a high rate of resistance to commonly used antimicrobial agents, including beta-lactams (penicillins and cephalosporins), fluoroquinolones, aminoglycosides and glycopeptides, which may reduce the effectiveness of these drugs.

Tigecycline is a new glycylcycline antibitiotic that has come into clinical use at a critical time and demonstrates *in vitro *activity against a wide range of bacteria [3,4]. Tigecycline has been approved by the Food and Drug Administration (FDA) and the European Medicines Agency (EMEA) for the treatment of complicated intra-abdominal and skin and soft tissue infections [5,6]. Besides, FDA recently approved it for the treatment of community-acquired pneumonia [7]. However, the role of tigecycline in the treatment of infections due to MDR bacteria remains undefined.

We conducted a prospective study to determine both clinical and microbiological outcomes of patients treated with tigecycline for serious hospital-acquired infections (HAI).

## Methods

The study was conducted at the San Martino Hospital, a 1500-bed, academic, tertiary care medical centre in Genoa (Italy). All adult subjects admitted to the hospital who received ≥48 h of treatment with tigecycline between January 1, 2007 and January 31, 2010 for treatment of HAI were enrolled. All patients received standard FDA and EMEA -approved dosage of tigecycline (initial loading dose of 100 mg, followed by 50 mg administered intravenously every 12 h). Identification and susceptibility testing of bacterial isolates were performed by standard techniques, with the use of a semi-automated system (Vitek 2; bioMerieux). Clinical data was collected from medical records and included age, sex, comorbidities, APACHE II score, clinical diagnosis, microbiologic isolate identification with antibiotic susceptibility, concomitant antibiotics, indication for tigecycline use, duration of tigecycline treatment and adverse clinical events.

Established criteria were used to define an hospital-acquired infection [8]. Secondary peritonitis was defined as a result of spillage of gut organisms through a physical hole in the gastrointestinal tract or through a necrotic gut wall and included intra-abdominal abscess (including liver, spleen and pancreatic), perforated diverticulitis complicated by abscess formation or fecal contamination; complicated cholecystitis with evidence of perforation, empyema, or gangrene; perforation of a gastric or duodenal ulcer; purulent peritonitis or peritonitis associated with fecal contamination; or perforation of the large or small intestine with abscess or fecal contamination; tertiary peritonitis was defined a peritonitis in a critically ill patient which persists or recurs at least 48 h after apparently adequate management of primary or secondary peritonitis. Empirical use of tigecycline was defined as the administration of treatment to a patient with signs and symptoms of infection without an identified source or a specific microbiological isolate. Targeted therapy was defined as the antibiotic administration in presence of an identified isolate.

Clinical response at the end of treatment was defined as positive response (partial or complete improvement of signs/symptoms of infection), negative response (no improvement or deterioration of signs/symptoms of infection), or uncertain response. Microbiological response was defined as positive response (sterile culture results during or after the course of antibiotic therapy), negative response (persistent identification of the same organism for 3 days after initiation of antibiotic treatment), or not documented response. The overall response was considered positive if any of the positive criteria of microbiological response were met.

The study was approved by the local institutional review board (Comitato Etico, Azienda Ospedaliera Universitaria San Martino, Genova, Italy) and patient consent was not required because of the observational nature of this study.

## Cases presentation

Two hundred and seven patients received tigecycline therapy for ≥ 48 h within our facility during the defined study period for the treatment of HAI. The clinical characteristics of the patients were reported in Table 1. Tigecycline was mainly used in the following wards: general surgery, oncology, haematology and intensive care. The therapy was targeted in 130/207 (63%) and empirical in 77/207 (37%) patients. All bacteria treated were susceptible to tigecycline. Median duration of tigecycline therapy was 13 days (range, 6-28) (table2). In table 2 type of infections and clinical efficacy of tigecycline are detailed. Table 3 details bacterial isolates and eradication rates. In 161/207 (78%) cases tigecycline was used as monotherapy, while 46/207 (22%) patients received tigecycline as part of a combination therapy, mainly associated with colistin (19/46; 41%), meropenem (11/46; 24%), amikacin (9/46; 20%), or ciprofloxacin (7/46; 15%). Clinical success was obtained in 151/207 (73%) cases, with the highest success rate recorded in intra-abdominal infections [81/99 (82%)]. Microbiological success was achieved in 100/129 (78%) treated patients. Adverse clinical events were seen in 16/207 patients (7.7%): 5 (2.4%) experienced mild nausea, and 12 (5.8%) experienced nausea and vomiting, while 7 (3.4%) experienced an increasing in liver enzymes. Only 4 (1.9%) of the total 207 patients experienced diarrhoea on tigecycline, all of whom were negative for C. difficile toxin in stools. Only one patient interrupted tigecycline in reason of adverse effects (profuse vomiting and nausea).

**Table 1:** Clinical characteristics of patients at start of tigecycline

	*n, 207*
Gender, *n *(%)	
Male	*118 (57)*

Age, yrs	
Median	*63*
Range	*14-89*

Apache II score	
Mean (± SD)	*21 ± 8.8*
Range	*8-45 8-45*

Admitted to ICU, *n *(%)	*83( 40)*

*Co-morbid conditions, n (%)*	
*Solid tumor*	*79(38)*
*Hematologic malignancy*	*50(24)*
*Diabetes mellitus*	*48(23)*
*Neutropenia (< 500 mm3)*	*29(14)*

**Table 2:** Type of infections, duration of treatment and clinical efficacy of tigecycline

Type of infections	*n *(%)	*Duration of treatment, days* *Median (range)*	Clinical efficacy *n *(%)	*Clinical failure* *n (%)*
Secondary peritonitis	46 (22)	9 (6- 18)	40 (88)	*6 (12)*
Tertiary peritonitis	41 (20)	15 ( 11-28)	32(78)	*9 (22)*
Other abdominal infections	12(6)	11 (7-17)	5 (42)	*7 (58)*
Pneumonia ( HAP, HCAP, VAP)	27 (13)	12 ( 8-21)	18 (67)	*9 (33)*
Pneumonia and bloodstream infections	29 (14)	17 (13-24)	19 (66)	*10 (34)*
Bloodstream infections	23 (11)	15 (12-18)	16 (70)	*7 (30)*
Complicated skin and soft tissue infections	17 (8)	11 (7-18)	13(76)	*4 (24)*
Empiric use in neutropenic	12 (6)	14 (9-17)	7 (58)	*5(42)*
***Total***	***207 (100)***		***151 (73)***	***56 (27)***

HAP: hospital acquired pneumonia, HCAP: health care associated pneumonia, VAP: ventilator associated pneumonia

**Table 3:** Bacterial isolates treated with tigecycline and eradication rate.

Bacterial isolates	*n *(%)	Microbiological efficacy *n *(%)	*Microbiological failure* *n(%)*
*Enterococcus faecium*	31 (24)	22/31 (71)	*9/31 (29)*
*Escherichia coli*	26 (20)	21/26 (81)	*5/26 (19)*
Methicillin-resistant *Staphylococcus aureus*	20 (15)	16/20 (80)	*4/20 (20)*
*Acinetobacter baumannii*	16 (12)	11/16 (69)	*5/16 (31)*
*Klebsiella pneumoniae*	15 (12)	13/15 (89)	*2/15 (11)*
*Enterobacter cloacae*	10 (8)	8/10 (80)	*2/10 (20)*
*Enterococcus faecalis*	6 (5)	6/6 (100)	*0/6*
Others	5 (4)	3/5 (60)	*2/5 (40)*
*Total*	*129*	*100/129 (78)*	*29/129(22)*

## Conclusions

We described 207 patients who received tigecycline under real-life conditions of daily clinical practice for empiric or targeted treatment of serious HAI. Tigecycline was used to treat a variety of infections, including many that were not indicated in official FDA and EMEA labelling for tigecycline. Despite the fact that most of the patients were critically ill and requiring ICU care or they had high APACHE II scores at the time of tigecycline administration, the overall clinical outcomes were good. The patients analyzed in this prospective study constitute the biggest relevant cohort. Successful clinical response rates of 82% were recorded for intra-abdominal infections and 78% for complicated skin and soft tissue infections with an overall successful clinical response of 73%. The mean clinical response rate was lower in patients with febrile neutropenia, pneumonia and bacteremia (58%, 67% and 70%, respectively). The population represented in this study included a significantly higher proportion of seriously ill patients which was not captured in the Phase 3 registration trials for each therapeutic indication. In a pooled analysis of two Phase 3 double-blind trials of tigecycline versus imipenem-cilastatin in patients with complicated intra-abdominal infection, the mean APACHE II score of tigecycline-treated patients was 6.3, and only 3.5% of them had an APACHE II score higher than 15; an APACHE II score of more than 20 was an exclusion criterion in those studies [5]. The population treated in our study mirrors the typical patients with complicated HAI and significantly higher APACHE II scores (mean score of 21) and thus higher disease severity and the data confirm similar recent experiences [9,10]. In addition, the majority of the patient population experienced co-morbidities leading to a higher risk of infections with MDR bacteria.

In approximately half of the patients, complicated intra-abdominal infections were involved, including MRSA and *Enterococcus spp *infections. We observed a remarkably high proportion of enterococcal infections and among them tigecycline had a success rate of 76%, similar to the microbiological efficacy obtained against MRSA (80%). Regarding the anti-Gram-negative efficacy, tigecycline confirms its effectiveness especially for *E. coli *(81%) and *A. baumannii *( 69%). This data confirms recent similar experiences reported [11-14]. The majority of patients received tigecycline in monotherapy and only 22% of patients were treated with tigecycline in combination with other broad-spectrum antibiotics to expand the range of activity against *P. aeruginosa*.

In this non-comparative trial, tigecycline appeared safe and effective in the treatment of serious HAI caused by resistant bacteria. The data from this study is consistent with larger pivotal studies of tigecycline treatment of serious infections [5-7]. For these reasons, tigecycline may be useful as an addition to the clinician's antimicrobial therapy options for difficult-to-treat resistant pathogens associated with serious nosocomial infections and also as part of an overall infection control and pharmacy intervention as suggested in the current guidelines [15].

The results should be viewed as purely descriptive, as this was an observational, prospective analysis of data from 207 patients who had received tigecycline in our hospital and no control group of patients (treated with drugs other than tigecycline) was analysed in this assessment. Considering the lack of data on newly approved antimicrobial agents for critically ill patients, we think that the reported data of our clinical experience with tigecycline, despite the limitations discussed can be useful for clinicians.

## Competing interests

Matteo Bassetti have received speaker's honoraria and/or research grant from: Pfizer, Astellas, MSD, Novartis, Bayer, Aventis, GSK and Astra Zeneca

The other authors declare that they have no competing interests

## Authors' contributions

MB: Data acquisition, data/statistical analyses, drafting the manuscript; LN: Sample collection, drafting the manuscript, critically revising for medical content; ER: Sample collection, critically revising for medical content; ER: Study design and conception, critically revising for medical content; VD: Data acquisition, data analyses; CV: Study design, conception and coordination; All authors contributed to writing of the final manuscript; All authors read and approved the final manuscript

## Pre-publication history

The pre-publication history for this paper can be accessed here:

http://www.biomedcentral.com/1471-2334/10/287/prepub
